# Abortion Doula Programs Across the United States: A Review of the Current Landscape

**DOI:** 10.7759/cureus.100501

**Published:** 2025-12-31

**Authors:** Caroline N Goldfarb, Caryn Dutton, Elizabeth Bastias-Butler, Chloe Zera, Sara Neill, Elysia Larson

**Affiliations:** 1 Obstetrics and Gynecology, Beth Israel Deaconess Medical Center, Boston, USA; 2 Obstetrics, Gynecology, and Reproductive Biology, Harvard Medical School, Boston, USA; 3 Family Planning, Beth Israel Deaconess Medical Center, Boston, USA; 4 Clinical Services, DuPont Clinic, Washington, D.C., USA

**Keywords:** abortion care, abortion doula landscape, abortion doula programs, abortion doulas, program evaluation

## Abstract

Abortion doulas support patients who are receiving abortion care. Understanding the abortion doula landscape is critical for patients, clinicians, doulas, and those interested in expanding programs for doula support. We aimed to describe the state of US-based abortion doula programs through a mapping review of the grey literature, documenting programs' characteristics from their online presence (websites, Facebook, Instagram, X). We identified 17 active abortion doula programs. In addition, we identified 30 programs which appeared either to be inactive or to not provide abortion support, primarily in states with post-Dobbs abortion bans. Most active abortion doula programs are community-based, volunteer-run, and free for patients. Three doula programs have partnerships with local clinics; three clinics have in-house doula programs. Most train their own doulas. Most US-based abortion doula programs independently provide community-based, no-cost, volunteer doula services.

## Introduction and background

Doulas provide physical, emotional, and informational support to people through major health experiences, most commonly labor and birth. Research demonstrates the benefits of birth doula support for improving patient satisfaction, reducing C-section rates, and mitigating healthcare inequities [1]. Community recognition of the need for support throughout all outcomes of pregnancy, including miscarriage and abortion, inspired broadening the birth doula model to a "full-spectrum" model. Full-spectrum doulas provide emotional, physical, and informational support to individuals through the full spectrum of reproductive experience, including birth, miscarriage, and abortion.

In addition to full-spectrum doulas, there are abortion-specific doulas that provide emotional, physical, and informational support to patients only through abortion care, including medication and procedural abortion care in both the community and clinic settings. In the clinic setting, abortion doula support may include meeting with patients before the procedure to answer questions, offer validating and reassuring emotional support, and help them prepare. During the procedure, doulas may coach patients through breathing techniques or visualization exercises, and afterward, they assist with comfort and care in the recovery area. In community settings, doulas can provide in-person support to help patients remain physically comfortable during and after abortion care, respond to any questions, and offer ongoing emotional support through discussion, validation, and other comforting interactions. Doulas may also provide virtual support, texting with patients throughout their abortion care to offer support and answer questions.

Doula support for abortion may be especially beneficial as patients' needs for physical and emotional support may be uniquely complicated by sociopolitical dynamics, stigma, and marginalization [1,2]. Abortion doulas practice through a range of models, including volunteer organizations that may or may not partner with clinics to provide in-clinic support, paid positions within clinics, volunteer collectives that offer only virtual services, and independent practices. Abortion doula support has been found in a few small randomized controlled trials and qualitative studies to be of interest to patients and clinic staff, with doulas reporting their role in providing patients' support and advocacy, especially through the complex web of abortion restrictions and stigma [1-7].

Despite increasing recognition, the landscape of abortion doula programs across the United States has not yet been described. One study described the landscape of abortion doulas across Canada and found that abortion doulas helped patients navigate complex and fragmented systems of medical care and practical support and provided support to patients experiencing marginalization and oppression [3]. The few US-based studies that have examined abortion doulas' impact were limited to pilots at academic medical centers, though they found patients were mostly interested in abortion doula care [1,4-7]. One randomized controlled trial comparing patients' experience between those who were and were not given doula support for their abortion care found patients who had doula support overwhelmingly approved of it (96.2%); the majority of those who had not been offered a doula desired one (71.6%). Of note, additional staff were needed to support patients who did not have doula support, demonstrating an additional benefit to abortion doula support for the distribution of staff time and resources [7]. A recent scoping review examined four studies and focused on outcomes related to patient experience and barriers to doula care; the review did not investigate the model of abortion doula care which was being evaluated in each of the included studies [4].

Several states have recognized the value of doulas in the birth setting, opting to cover childbirth doula programs as a population-level strategy to decrease maternal morbidity and mortality [2]. To fulfill the aims of reproductive justice, the right to bodily autonomy (to have children when desired, to not have children, and to parent our children in safe and sustainable communities [8]), we must support patients not only as they seek to have children but also as they seek to end their pregnancies. As abortion care becomes increasingly politicized, fragmented, and criminalized, abortion doulas can help patients navigate the complexities of seeking abortion care while providing patients with direct, one-on-one support, helping fight inequities in access and experience of care, especially those which are exacerbated by geographic and political barriers [9]. Abortion doula organizations can help improve patient support, facilitate complex care coordination, and empower individuals' bodily autonomy, building towards the future of reproductive justice [1-5,9]. This mapping review is a critical step towards outlining potential models for doing just that through understanding the state of active abortion doula programs across the United States.

We aimed to describe the state of the US abortion doula program landscape to help better understand the models of care for patients and providers looking for available support and for organizations and others seeking to expand abortion doula access.

Some information from this article was previously presented as an abstract at the National Abortion Federation Annual Meeting in Washington, D.C., in 2024.

## Review

Methods

To provide an up-to-date review of active abortion doula programs across the United States, we conducted a mapping review of the grey literature, information available online and on social media (Google, Facebook, Instagram, X). A mapping review differs from scoping and systematic reviews by focusing on cataloging and categorizing existing literature on a topic rather than evaluating study quality or synthesizing multiple studies' findings in depth.

The search was conducted in July of 2024. The search strategy included Google searches with terms "abortion doula programs", "find an abortion doula", and "abortion clinics with doulas". We investigated each link on the first four pages of each Google query for relevance. If a link led to an abortion doula website, we included that program in screening. Inclusion criteria was any website or social media (Facebook, Instagram, X) which either explicitly described providing abortion doula support or described providing full-spectrum doula support. Exclusion criteria was any website or social media page which was not for a doula organization or collective and/or did not mention abortion or full-spectrum doula support. Our first search also surfaced a list of doula organizations from a co-developer of the full-spectrum doula model's website, Radical Doulas; we screened each organization on that list [10].

Next, we investigated each program to determine its active status. We deemed a program "active" if it had website or social media posts within the past year, unless specifically declared inactive. For active programs, we collected specific characteristics including provider partnerships, in-person versus virtual support options, and doula and patient payment structures.

In summary, we conducted a mapping review of the grey literature, which included Google, X, Facebook, and Instagram; programs were screened if they mentioned abortion doula support and were further classified as active (posts on these sits within the last 12 months) or inactive (no posts within the last 12 months or declaration of a program's inactive status).

Results

**Figure 1:**
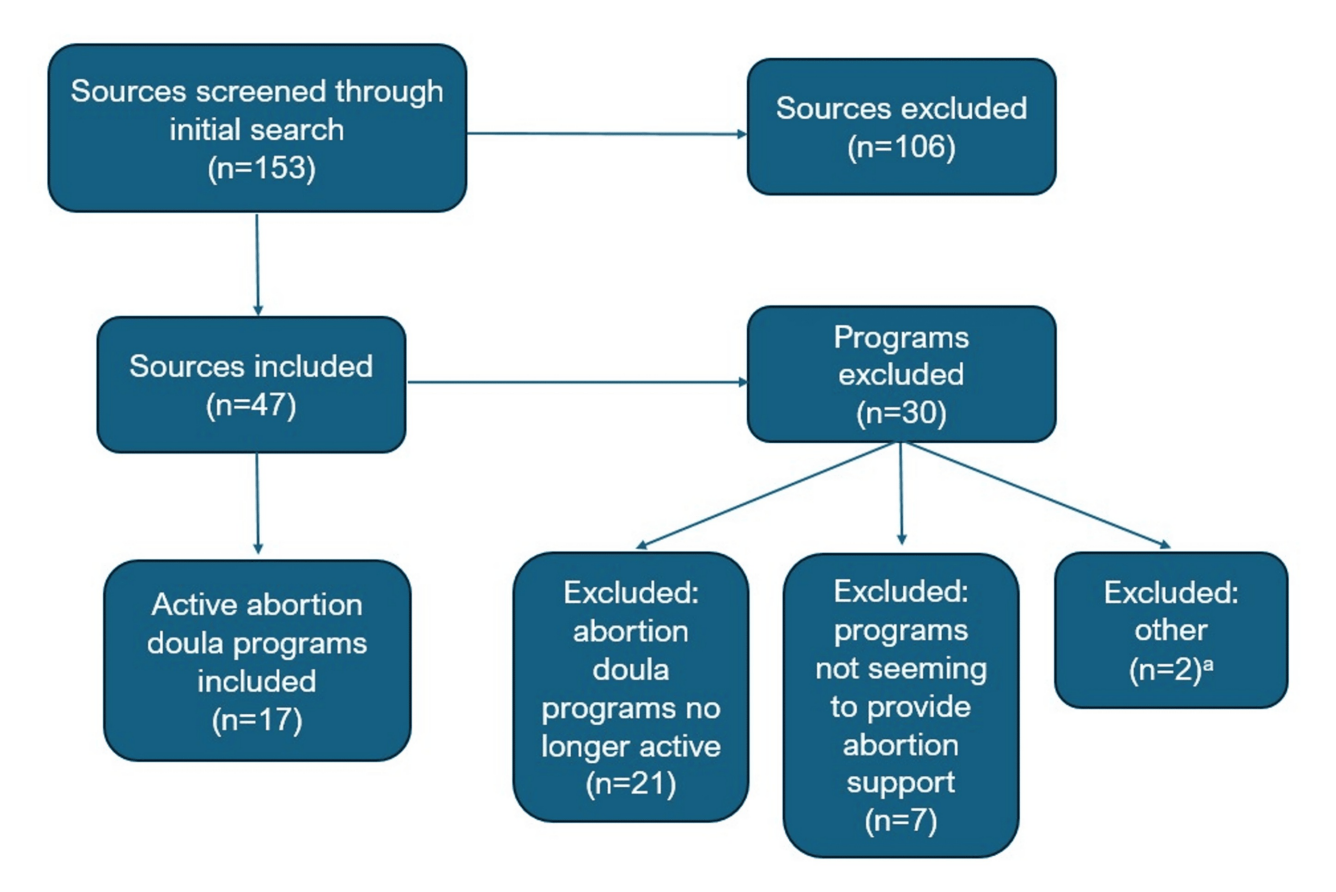
Data selection for abortion doula programs across the United States ^a^One program was excluded because it appeared to be a solo-practitioner's website. One program was excluded because it was a list of state-registered doulas. This figure describes abortion doula programs identified in the United States identified via a scoping review in 2024.

We screened 153 sources: 120 from Google queries and 33 from the Radical Doulas website. We identified 47 total abortion doula programs. Of these, we excluded 30 programs which were wholly inactive or not providing abortion support (Figure 1).

We identified 17 active US-based abortion doula programs [11-27] (Table 1).

**Table 1:** Program characteristics of 17 active abortion doula programs in the United States ΔTotals do not sum to 100 due to programs with unclear responses; for example, for two programs, it was unclear what type of support was provided. *All Southern programs were in Division 5, the South Atlantic. This table describes the characteristics of active abortion doula programs in the United States found in 2024.

	N	%
Type of support Δ		
Only virtual	2	(13%)
Only in-person	6	(40%)
Both virtual and in-person	7	(47%)
Patient payment		
Free	14	(82%)
Unclear	3	(18%)
Association with clinic		
Independent	11	(65%)
Affiliated	3	(18%)
Embedded	3	(18%)
Doula payment		
Volunteer	13	(76%)
Paid	1	(6%)
Unclear or stipend	3	(18%)
Doula training		
Program provides training	16	(94%)
Unclear	1	(6%)
Training cycle Δ		
Annual	7	(70%)
Bi-annual	2	(20%)
Online anytime	1	(10%)
Region		
West	6	(35%)
Midwest	2	(12%)
South*	5	(29%)
Northeast	3	(18%)
N/A (solely online)	1	(6%)

Active programs spanned 10 states and Washington, D.C.; one is solely online (Table 2).

**Table 2:** Program-specific characteristics of 17 active abortion doula programs in the United States *In all columns: 0=no, 1=yes, and 2=unclear SPIRAL: Supporting People in Reproduction, Abortion, and Loss; WISE: Women In Support and Empowerment This table describes the characteristics of active abortion doula programs in the United States found in 2024.

Program	State	US census region	Partners with a clinic?	Type of support provided?				Is it free for patients?	Do they provide their own training?	Do patients find them online?	Are doulas volunteers?
				Medication abortion	Waiting room	Virtual	In-person				
PP AZ [11]	AZ	West	1	1	2	0	1	1	1	0	1
Los Angeles Support Collective [12]	CA	West	0	1	1	1	1	1	1	1	1
Birthworkers of Color Collective [13]	CA	West	0	1	1	1	1	1	1	1	0
The Doula Project [14]	NY	Northeast	0	1	0	1	0	1	1	1	1
Wesleyan Doula Project [15]	CT	Northeast	1	0	1	0	1	1	1	0	1
Dopo [16]	n/a (virtual only)	n/a	0	1	2	1	2	1	1	1	1
DC Doulas for Choice [17]	DC	South Atlantic	1	1	2	2	1	1	1	0	1
DuPont Clinic [18]	DC	South Atlantic	0	0	1	0	1	1	1	0	0
Colorado Doula Project [19]	CO	West	0	1	1	1	1	1	1	1	1
Mountain Area Abortion Doula Collective [20]	NC	South Atlantic	0	1	0	1	0	1	1	1	1
Baltimore Doula Project [21]	MD	South Atlantic	1	2	2	2	1	1	1	1	1
Bay Area Doula Project [22]	CA	West	0	1	1	1	1	2	1	1	1
SPIRAL Collective [23]	MN	Midwest	0	1	1	1	1	1	1	1	1
Ancient Song Doula Services [24]	NY	Northeast	0	0	1	0	1	2	1	1	0
WISE Community Doulas [25]	NC	South Atlantic	0	2	2	2	1	2	1	1	0
Planned Parenthood Southwest Ohio [26]	OH	Midwest	1	0	2	0	1	1	1	0	1
Cascades Abortion Support Collective [27]	OR	West	0	1	0	1	1	1	2	1	1

Program Models

Most active abortion doula programs are independent, community-based organizations led and served by volunteer doulas (n=14) (Table 1). In this model, doulas are contacted directly by patients seeking support. Some have clinic partnerships (n=3). These programs mostly provide support to patients throughout medication abortion while the patient is home and/or to patients before and after procedural abortion. Some programs explicitly offer support in clinic waiting rooms; none mention offering support in the procedure room.

In contrast to the community-based model described above, three programs are housed within abortion clinics. Rather than being contacted directly, these doulas serve shifts at clinics where patients are offered doula support for their care. Of these, one is a paid program; two others are volunteer.

Seven programs mention offering support virtually and in-person. Six programs offer only in-person support; two offer only virtual support; the rest were unclear. For in-clinic support, eight programs mention supporting patients in clinic waiting rooms, while only three programs provide patients with support during the abortion procedure.

Training Structures

We also examined the training and other services provided by these programs. Almost all doula programs offer their own abortion doula training and onboard their trained doulas on a regular yearly or bi-annual basis. Some groups (4/17; 24%) also provide patients with practical support like assistance with transportation, lodging, and food. Groups providing practical support described intake processes whereby patients describe their needs to program volunteers via an online form or phone call.

Geographic Distribution

Turning to inactive programs excluded from further review, 38% (8/21) were in states with post-Dobbs abortion bans [28]. One inactive program explicitly attributed its closure to the Supreme Court ruling in Dobbs v. Jackson Women's Health Organization (2022). 

All programs were identified through an online search. Prior work has shown that online searches can be a viable source of self-referral for abortion services [29], and the present work shows the same to be true for self-referral for abortion doula services.

## Conclusions

We found that abortion doula organizations support people across the country, providing emotional, physical, and informational support both in the clinic and at home. Abortion doula programs are mostly community-based and led and run by volunteers. Patients can find them online, via social media, practical support organizations, and abortion funds. Few organizations have clinic partnerships to provide in-clinic doula support. Of the three clinics with doula programs, only one was found to have paid doulas staffed in-house. Other programs have shut down in states with abortion bans, though few publicly attribute their closure to the Supreme Court ruling in Dobbs v. Jackson. 

One limitation for this review is the grey-literature methodology, as having access to only publicly available online information may lead to our data being out-of-date, incorrect or misleading, or otherwise being variable in quality. Further, there are many reasons why abortion doula programs may choose to not have a public online presence, including fear of legal retaliation in restrictive states, fear of protestors and other anti-abortion tactics, and desire to protect marginalized clients. However, given that this is the same information available to anyone seeking information online for the purposes of self-referral this review thus represents an accurate portrayal of what individuals may find when looking into abortion doula support.

Future research should examine the factors impacting abortion doula support programs, including causes of program closures, impact of virtual support, and doulas’ views on program sustainability.

While informal support throughout pregnancy loss and termination are age-old practices, formalized abortion doula programs are emerging in the U.S. and constitute a diverse and ever-changing patient support landscape. While some research has described the outcomes of pilot programs in academic medical center settings or the landscape of grassroots' abortion funds, this review of the state of abortion doula programs goes further by examining how abortion doula support is provided on a larger scale. This review identifies characteristics of active abortion doula programs and provides key information for those interested in sharing information about available options with patients, those seeking to develop new abortion doula programs, and those considering policy aimed at supporting and expanding abortion doula care.
